# Use of glucocorticoids and risk of breast cancer: a Danish population-based case-control study

**DOI:** 10.1186/bcr3106

**Published:** 2012-02-03

**Authors:** Gitte Vrelits Sørensen, Deirdre P Cronin-Fenton, Henrik Toft Sørensen, Sinna Pilgaard Ulrichsen, Lars Pedersen, Timothy L Lash

**Affiliations:** 1 Department of Clinical Epidemiology, Aarhus University Hospital, Olof Palmes Allé 43-45, 8200 Aarhus N, Denmark

## Abstract

**Introduction:**

Glucocorticoids are widely prescribed drugs. In the human body, glucocorticoid is the main stress hormone and controls a variety of physiological and cellular processes, including metabolism and immune response. It belongs to the same steroid superfamily as estrogens, which are known to play a role in breast cancer. However, the effect of glucocorticoid use on the risk of breast cancer is not clear.

**Methods:**

We conducted a case-control study using population-based medical databases from Northern Denmark (1.8 million inhabitants) to investigate the association between glucocorticoid prescriptions and breast cancer risk. The study included 9,488 incident breast cancer cases diagnosed between 1994 and 2008 and 94,876 population controls. We estimated the odds ratios (ORs) and 95% confidence intervals (CIs) associating glucocorticoid use with breast cancer occurrence, controlling for prescriptions of postmenopausal hormone replacement therapy, anti-diabetics, immunosuppressive drugs, and hospital diagnosis of obesity, diabetes, chronic pulmonary diseases and autoimmune diseases.

**Results:**

We found no effect on breast cancer risk in ever users (> 2 prescriptions) of any glucocorticoids (adjusted odds ratio (aOR) = 1.0; 95% CI: 0.96, 1.1), systemic glucocorticoids (aOR = 1.0; 95% CI: 0.96, 1.1), or inhaled glucocorticoids (aOR = 1.0; 95% CI: 0.95, 1.1), each compared to never users of any glucocorticoids. Associations for recent use (preceding two years) and former use (more than two years earlier) were near null in all dose categories (low, medium and high number of prescriptions). Intensity of systemic glucocorticoid use (cumulative prednisolone equivalent doses), regardless of duration (< 1, 1 to 5, 5+ years), was also not associated with breast cancer risk.

**Conclusions:**

Overall, our study provides no evidence that glucocorticoid use affects the risk of breast cancer.

## Introduction

Glucocorticoid is an adrenocortical hormone, belonging to the same steroid superfamily as estrogen, which is known to play a role in breast cancer risk [1]. Glucocorticoids mediate most of their effects through the intracellular glucocorticoid receptor (GR), which subsequently modulates downstream gene regulation [2]. The GR is expressed in breast tissue and has been shown to be involved in normal breast development during pregnancy [3]. In the human body, glucocorticoid is the main stress hormone and controls a variety of physiological and cellular processes including metabolism, cell growth, apoptosis, and immune response [2]. Thus, it could potentially play a role in the risk of breast cancer by several mechanisms.

Synthetic glucocorticoids affect immune function and are commonly used as anti-inflammatory and immunosuppressive therapy in diseases such as asthma, chronic obstructive pulmonary disease (COPD), inflammatory bowel disease (IBD), inflammatory arthritis, and other connective tissue disorders [4]. The immune system has a role in preventing cancer development and progression [5,6], so suppression of the immune system may promote tumor development. However, the role of the immune system in cancer is dual and complex, since it may also promote tumor growth [5]. In addition, decreased glucose tolerance, a well-known side effect of glucocorticoids [4], has been suggested to promote mammary carcinogenesis [7-9].

To our knowledge, only one study has previously been published on the relation between glucocorticoid use and breast cancer risk [10]. Our previous study included nearly 33,000 women, ascertained prescriptions for systemic glucocorticoids between 1989 and 1996, ascertained breast cancers until 1998, and recorded a total of 367 breast cancer cases. We reported no association between glucocorticoid prescriptions and breast cancer risk. Due to a relatively short average follow-up time (mean 5.8 years), we were not able to investigate the effect of long-term use. Also, we were only able to control for age as a potential confounder.

A recent review called for further research investigating the potential association between glucocorticoid use and breast cancer risk, criticizing our previous study for lack of clinical details and the possibility of confounding [11]. We, therefore, conducted a large population-based case-control study with prospectively collected prescription data to examine the association between glucocorticoid use and breast cancer risk. In addition to following a larger population over a longer period, we have extended our previous study [10] by incorporating more information on potential confounders, such as parity, age at first birth, use of postmenopausal hormone replacement therapy (HRT), anti-diabetic medicine, immunosuppressive drugs, any history of hospital diagnosed obesity, diabetes, COPD, asthma, rheumatoid arthritis (RA), IBD and other autoimmune diseases (see Additional file 1).

## Materials and methods

### Source population

We conducted this population-based case-control study among female residents of the Central and North Denmark Regions between 1 February 1994 and 31 December 2008. Together, these regions represent about one-third of the Danish population (approximately 1.8 million inhabitants). The Danish National Health Service provides tax-supported health-care to all residents of the country and refunds part of the patients' expenditures on most physician-prescribed drugs, including glucocorticoids [12].

All health-related services are registered to individual patients by use of their civil personal registration (CPR) number, assigned to all Danish citizens since 1968 by the Danish Civil Registration System (CRS). This number encodes gender and date of birth [13] and facilitates accurate individual-level linkage between population-based registries.

The Danish regions are served by pharmacies equipped with computerized accounting systems through which data are sent to a regional prescription database hosted by Aarhus University [12], with key information about prescriptions for refundable drugs. Thus, the prescription database includes information on each patient's CPR number; the type and quantity of drug prescribed according to the Anatomical Therapeutic Chemical classification system (ATC), and the date the prescription was filled [12,14]. The Danish regions were until 1 January 2007 divided into counties. Because the different counties started contributing data to the prescription database at different times, they differ with respect to the earliest availability of prescription data, with the earliest being 1989 [12]. To ensure that we had at least minimal prescription data history for each case and their corresponding controls, we only included women who had more than 5 years of prescription history before their index date (as defined below). Women who were resident in the study area for less than five years before index date were excluded from the source population, as were women with any malignant cancer diagnosis before their index dates (except non-melanoma skin cancers).

### Cases

The sample size was determined by using the Danish Cancer Registry (DCR) to identify all women with an incident diagnosis of breast cancer in the region during the study period (see Additional file 1 for International Classification of Diseases (ICD) codes). The DCR has recorded all incident cancers through 31 December 2008 [15] and has near 100% completeness for breast cancer diagnosis [16]. With this sample size, our study had 90% power to detect an odds ratio of 1.1 or greater.

### Population controls

Controls were identified using the CRS [13,17]. A pool of eligible individuals with the same birth year and county/region of residence as each breast cancer patient was sampled from the general-population among all women alive and free of breast cancer on the date of the matched case's breast cancer diagnosis. This date of diagnosis, therefore, served as the index date for the case and all of the controls matched to the case. Ten individuals from this pool were randomly selected for each case. All eligible controls were included when the risk-set of matched controls included fewer than ten individuals.

### Data collection

The prescription database [12] was used to identify all prescriptions before index date for systemic (oral and injected), inhaled and local-acting glucocorticoids with intestinal anti-inflammatory effect ('local glucocorticoids' hereafter). The following preparations were available and prescribed during the study period (see Additional file 1 for ATC codes): betamethasone, dexamethasone, methylprednisolone, prednisolone, prednisone, triamcinolone, cortisone, hydrocortisone, beclomethason, budesonide, flunisolide, fluticasone, and mometasone.

### Potential confounding prescription drugs

The prescription database also provided information on use of the following potential confounding drugs before the index date (see Additional file 1 for ATC codes): postmenopausal HRT [18], anti-diabetics [19], and use of immunosuppressive drugs as a marker of immune-related disease.

### Potential confounding diseases

The Danish National Patient Registry (DNPR) contains information about all non-psychiatric hospital admissions since 1977, and since 1995 also outpatient visits [20]. From the DNPR we retrieved data on hospital diagnoses of the following diseases before the index date: obesity, diabetes, COPD, asthma, RA, IBD (ulcerative colitis or Crohn's disease), and a list of 'other autoimmune diseases' (see Additional file 1 for the list of diseases and associated ICD codes).

### Other potential confounders

To obtain information about parity and age at first birth we used the CRS [13]. In the CRS, the CPR numbers of parents are linked to their child, as long as the child lived with the parents in 1968 or later. We limited the analysis adjusted for parity and age at first birth to women born later than 1949, for whom the CRS allows reconstruction of childbearing history with better than 95% completeness [21].

### Analytic variables

We categorized glucocorticoid use as never versus ever use. Ever users were further subdivided into two dose categories: one or two prescriptions or more than two prescriptions. We examined the association of overall glucocorticoid use with breast cancer and then examined the association of systemic and inhaled glucocorticoid use separately. To examine the temporality of glucocorticoid use and the risk of breast cancer, we divided ever use into recent use (only prescriptions within two years of the index date) and former use (prescriptions earlier than two years of the index date). In the group of any glucocorticoid users we further divided the number of prescriptions into low (one or two prescriptions), medium (three to nine) and high (more than nine). We based these cut-offs on the distribution of prescription counts among the controls with more than two prescriptions.

To examine whether breast cancer risk was associated with the intensity and duration of glucocorticoid use, we restricted the analyses to systemic glucocorticoid users and calculated prednisolone equivalent cumulative doses based on the equivalency table in Kelly's *Textbook of Rheumatology *[22] and grouped into the four categories: < 200 mg, 200 to 399 mg, 400 to 999 mg and ≥ 1,000 mg (for calculations see Additional file 2). The duration was defined as the number of days from the date of the first prescription to the date of the last prescription, plus the duration of the last prescription (estimated to be on average 30 days). We divided the duration of glucocorticoid use into short-term (less than one year), medium-term (one to five years), and long-term (more than five years).

The covariates ever use of immunosuppressive drugs, ever use of postmenopausal HRT, COPD, asthma, IBD, obesity, and RA were coded individually as dichotomous variables. Other autoimmune diseases (see Additional file 1) were merged into a dichotomous variable. Since diabetes and metformin use may have counteracting effects on breast cancer risk [19], we coded diabetes and anti-diabetic medicine use as a categorical variable with three possible levels: no diabetes or use of anti-diabetic medicine, diabetes or use of anti-diabetic medicine with ever use of metformin, and diabetes or use of anti-diabetic medicine without ever use of metformin.

### Statistical analysis

We computed the frequency and proportion of cases and controls within categories of demographic variables and covariates.

In all analyses, we used never use of any glucocorticoids as the reference group. For the use of any glucocorticoids, systemic glucocorticoids, and inhaled glucocorticoids, we stratified by all covariates and calculated crude and stratum-specific odds ratios (ORs) to evaluate potential confounding and effect measure modification. We used conditional logistic regression analysis to compute crude and adjusted ORs (aORs) and their associated confidence intervals (95% CI), with simultaneous adjustment for the use of postmenopausal HRT, immunosuppressive drugs, hospital diagnosed obesity, diabetes (plus/minus a history of metformin use), COPD, asthma, IBD, RA and 'other autoimmune disease.' Since we used risk set sampling of controls, the ORs are unbiased estimates of the corresponding incidence rate ratio in the underlying population [23]. We report estimates of association stratified by age categories (< 45, 45 to 55 and > 55, as estimated ranges for pre-, peri- and post-menopausal status). In the group of women < 45, we were able to make additional adjustment for parity and age at first birth, using the combined variable (see Table 1).

**Table 1:** Frequency distribution of cases and matched population controls in North Jutland County between 1994-2008, Aarhus County between 2001-2008, and Viborg- and Ringkjøbing Counties between 2003-2008

Characteristics	Cases (*N *= 9,488)		Controls^a ^(*N *= 94,876)	
	N	%	N	%
**Age at index date^a^**				
< 45	899	9.5	9,072	9.6
45 to 55	1,932	20	19,108	20
> 55	6,657	70	66,696	70
**County at index date^a, b^**				
North Jutland	4,424	47	44,236	47
Aarhus	3,168	33	31,680	33
Viborg	931	10	9,310	10
Ringkjøbing	965	10	9,650	10
**Calendar year at index date^a^**				
1994-1998	1,253	13	12,530	13
1999-2003	2,910	31	29,100	31
2004-2008	5,325	56	53,246	56
**Use of any glucocorticoids**				
Never	6,692	71	68,064	72
≤ 2 prescriptions ever	1,452	15	13,891	15
> 2 prescriptions ever	1,344	14	12,921	14
**Use of other immunosuppressive drugs**				
Never	9,395	99	93,880	99
Ever	93	1.0	996	1.0
**Use of postmenopausal hormone replacement therapy**				
Never	7,252	76	76,919	81
Ever	2,236	24	17,957	19
**Hospital diagnosed diabetes or ever use of anti-diabetic medicine**				
Never	8,961	94	90,180	95
Yes & never metformin	312	3.3	2,655	2.8
Yes & ever metformin	215	2.3	2,041	2.2
**Hospital diagnosed chronic obstructive pulmonary disease**				
No	9,093	96	91,161	96
Yes	395	4.2	3,715	3.9
**Hospital diagnosed asthma**				
No	9,266	98	92,583	98
Yes	222	2.3	2,293	2.4
**Hospital diagnosed inflammatory bowel disease**				
No	9,407	99	94,117	99
Yes	81	0.9	759	0.8
**Hospital diagnosed rheumatoid arthritis**				
No	9,392	99	93,733	99
Yes	96	1.0	1,143	1.2
**Other autoimmune diseases^c^**				
No	9,086	96	91,201	96
Yes	402	4.2	3,675	3.9
**Hospital diagnosed obesity**				
No	9,240	97	92,512	98
Yes	248	2.6	2,364	2.5
**Age at first birth in women born later than 1949 (*n *= 31,130)**^d^				
Nulliparous	344	12	3,373	12
< 25	1,284	45	13,845	49
25-30	885	31	8,345	30
> 30	317	11	2,737	10
**Parity in women born later than 1949 (*n *= 31,130)^d^**				
Nulliparous	344	12	3,373	12
1 child	445	16	4,170	15
2 children	1,341	47	13,005	46
≥ 3 children	700	25	7,752	27
**Combined parity & age (year) at first birth in women born later than 1949 (*n *= 31,130)^d^**				
Nulliparous	344	12	3,373	12
1 child & < 25 y	154	5.4	1,444	5.1
2 children & < 25 y	658	23	6,956	25
≥ 3 children & < 25 y	472	17	5,445	19
1 child & ≥ 25 y	291	10	2,726	9.6
2 children & ≥ 25 y	683	24	6,049	21
≥ 3 children & ≥ 25 y	228	8.1	2,307	8.2

^a^Controls were matched to cases on county/region of residence and birth year. ^b^The counties were merged into the two regions in 2007. Thus, from 2007 to 2008 the women were instead matched on former county. The higher proportion of people in North Jutland County was a result of longer prescription history. ^c^See Additional file 1 for the list of hospital-diagnosed other autoimmune diseases. ^d^To assure complete data on every birth of the woman we made a subgroup of women born later than 1949 (*n *= 31,130).

We used Stata 11.0 (StataCorp LP, College Station, TX, USA) and SAS 9.2 (SAS Institute Inc., Cary, NC, USA) for the data analyses. This study was approved by the Danish Data Protection Agency (reference #2004-41-4693). According to Danish law, registry-based studies such as this one do not require further approval by an ethical review board or informed consent by the members of the study population.

## Results

We identified 9,488 breast cancer cases and 94,876 population controls. Median age at the index date was 62.1 years. Characteristics of cases and controls are shown in Table 1. As expected, a higher proportion of cases than controls had ever used postmenopausal HRT by their index date (24% versus 19%). In the subgroup of women born later than 1949 (*n *= 31,130), a lower proportion of cases than controls had a first birth before age 25 (45% versus 49%) and had three or more children (25% versus 27%). In all other characteristics, there were no important differences between cases and controls.

We found no effect on breast cancer risk in ever users (> 2 prescriptions) of any glucocorticoids compared to never users (aOR = 1.0; 95% CI: 0.96, 1.1). The association among former users with a high number of prescriptions (> 9) was null (aOR = 1.0; 95% CI: 0.93, 1.1). In addition, among recent users who only had prescriptions within the two years before their index date, we found null associations. Restricting the analysis to systemic or inhaled glucocorticoid use also showed no associations. Ever use (> 2 prescriptions) of systemic (aOR = 1.0; 95% CI: 0.96, 1.1) or inhaled glucocorticoids (aOR = 1.0; 95% CI: 0.95, 1.1), each compared to never use of any glucocorticoids, were both null (Table 2). Intensity of systemic glucocorticoid use (cumulative prednisolone equivalent doses), regardless of duration (< 1, 1 to 5, 5+ years), showed no pattern of association (Table 3). Stratifying into estimates of pre-, peri-, and post-menopausal status by age group also did not show any pattern of association. In the subgroups of women < 45 years old (Table 4) and all women born later than 1949 (*n *= 31,130), additional adjustment for parity and age at first birth did not change our overall estimates of null association. In the latter group, the aOR for > 2 glucocorticoid prescriptions ever, compared to never, was 1.0 (95% CI 0.88, 1.2).

**Table 2:** Number of prescriptions (pres.) and temporality of any glucocorticoid (GC) use, systemic GC use and inhaled GC use and odds ratio of breast cancer

Characteristics	Cases (*N *= 9,488)		Controls^a^ (*N *= 94,876)		Odds Ratio^b^	95% Confidence Interval
	N	%	N	%		
**Any GC use**						
Never use	6,692	71	68,064	72	reference	reference
Ever use						
≤ 2 pres.	1,452	15	13,891	15	1.0	0.98, 1.1
> 2 pres.	1,344	14	12,921	14	1.0	0.96, 1.1
Recent use^c^						
≤ 2 pres.	284	3.0	2,949	3.1	0.96	0.84, 1.1
3 to 9 pres.	72	0.8	781	0.8	0.93	0.73, 1.2
> 9 pres.	13	0.1	156	0.2	0.82	0.46, 1.4
Former use^d^						
≤ 2 pres.	1,168	12	10,942	12	1.1	0.99, 1.1
3 to 9 pres.	655	6.9	6,142	6.5	1.0	0.96, 1.1
> 9 pres.	604	6.4	5,842	6.2	1.0	0.93, 1.1
**Systemic GC use**						
Never use of any GC	6,692	71	68,064	72	reference	reference
Only inhaled/local GC use	451	4.8	4,401	4.6	1.0	0.93, 1.1
Ever use of systemic GC						
≤ 2 pres.	1,437	15	13,719	15	1.0	0.98, 1.1
> 2 pres.	908	10	8,692	9.1	1.0	0.96, 1.1
Recent systemic use^c^						
≤ 2 pres.	278	2.9	2,903	3.1	0.95	0.84, 1.1
> 2 pres.	64	0.7	705	0.7	0.91	0.71, 1.2
Former systemic use^d^						
≤ 2 pres.	1,159	12	10,816	11	1.1	0.99, 1.1
> 2 pres.	844	8.9	7,987	8.4	1.0	0.96, 1.1
**Inhaled GC use**						
Never use of any GC	6,692	71	68,064	72	reference	reference
Only systemic/local GC use	1,886	20	17,951	19	1.0	0.98, 1.1
Ever users of inhaled GC						
≤ 2 pres.	317	3.4	3,223	3.4	1.0	0.87, 1.1
> 2 pres.	593	6.2	5,638	6.0	1.0	0.95, 1.1
Recent inhaled use^c^						
≤ 2 pres.	84	0.9	770	0.8	1.1	0.86, 1.4
> 2 pres.	38	0.4	453	0.5	0.83	0.59, 1.2
Former inhaled use^d^						
≤ 2 pres.	233	2.5	2,453	2.6	0.94	0.82, 1.1
> 2 pres.	555	5.8	5,185	5.5	1.1	0.97, 1.2

^a^Controls were matched to cases on county of residence and birth year. ^b^Analysis adjusted for any use of postmenopausal hormone replacement therapy or 'other immunosuppressive drugs' before index date, and any hospital diagnosis of obesity, diabetes (+/- metformin use), chronic obstructive pulmonary disease, asthma, inflammatory bowel disease, rheumatoid arthritis or 'other autoimmune disease' before index date. ^c^Recent use: gucocorticoid use only within two years of diagnosis, and never a former user. ^d^Former use: Glucocorticoid use earlier than within two years of diagnosis.

**Table 3:** Duration and intensity of systemic glucocorticoid (GC) cumulative doses in milligram (mg) prednisolone equivalent doses^a ^and odds ratio of breast cancer

Characteristics	Cases (*N *= 9,488)		Controls^b^ (*N *= 94,876)		Odds Ratio^c^	95% Confidence Interval
	N	%	N	%		
**Never any GC use**	6,692	71	68,064	72	reference	reference
**Only inhaled/local use**	451	4.8	4,401	4.6	1.0	0.93, 1.1
**Short-term (< 1 year of use)^d^**						
< 200 mg	1,237	13	11,830	12	1.0	0.97, 1.1
200 to 399 mg	94	0.99	990	1.0	0.93	0.75, 1.2
400 to 999 mg	21	0.22	242	0.26	0.88	0.57, 1.4
≥ 1000 mg	15	0.16	93	0.10	1.6	0.90, 2.8
**Medium-term (1 to 5 years of use)^d ^**						
< 200 mg	289	3.1	2,805	3.0	1.0	0.89, 1.2
200 to 399 mg	138	1.5	1,166	1.2	1.2	0.97, 1.4
400 to 999 mg	67	0.71	545	0.57	1.2	0.93, 1.6
≥ 1000 mg	20	0.21	273	0.29	0.73	0.46, 1.2
**Long-term (> 5 years of use)^d^**						
< 200 mg	170	1.8	1,683	1.8	1.0	0.85, 1.2
200 to 399 mg	123	1.3	1,141	1.2	1.1	0.87 1.3
400 to 999 mg	96	1.0	1,053	1.1	0.89	0.72, 1.1
≥ 1000 mg	75	0.79	590	0.62	1.2	0.96, 1.6

^a^For calculations see Additional file 2. ^b^Controls were matched to cases on county of residence and birth year. ^c^Analysis adjusted for any use of postmenopausal hormone replacement therapy or 'other immunosuppressive drugs' before index date and any hospital diagnosis of obesity, diabetes (+/- metformin use), chronic obstructive pulmonary disease, asthma, inflammatory bowel disease, rheumatoid arthritis or 'other autoimmune disease' before the index date. ^d^Duration was calculated as time between the first and the last systemic glucocorticoid prescription (plus 30 days from the last prescription), and then divided into short-, medium- and long-term use.

**Table 4:** Any glucocorticoid use and odds ratio of breast cancer stratified by age group as a conservative estimate of menopausal status

Characteristics	Cases (*N *= 9,488)		Controls^a ^(*N *= 94,876)		Odds Ratio^b^	95% Confidence Interval
	N	%	N	%		
**Age < 45 (pre-menopausal)**						
Never use	744	83	7,353	81	reference	reference
Ever use						
≤ 2 prescriptions	98	11	1000	11	0.97^c^	0.78, 1.2^c^
> 2 prescriptions	57	6.4	719	7.9	0.83^c^	0.61, 1.1^c^
**Age 45 - 55 (peri-menopausal)**						
Never use	1,488	77	14,450	76	reference	reference
Ever use						
≤ 2 prescriptions	248	13	2,757	14	0.86	0.75, 0.99
> 2 prescriptions	196	10	1,901	10	0.96	0.80, 1.1
**Age > 55 (post-menopausal)**						
Never use	4,460	67	46,261	69	reference	reference
Ever use						
≤ 2 prescriptions	1,106	17	10,134	15	1.1	1.0, 1.2
> 2 prescriptions	1,091	16	10,301	15	1.1	0.98, 1.1

^a^Controls were matched to cases on county of residence and birth year. ^b^Analysis was adjusted for any use of postmenopausal hormone replacement therapy or 'other immunosuppressive drugs' before index date and any hospital diagnosis of obesity, diabetes (+/- metformin use), chronic obstructive pulmonary disease, asthma, inflammatory bowel disease, rheumatoid arthritis or 'other autoimmune disease' before the index date. ^c ^Analyses were additionally adjusted for parity and age at first birth. Without the additional adjustment the adjusted odds ratios for ≤ 2 or > 2 prescriptions were 0.97 (95% CI: 0.77, 1.2) and 0.83 (95% CI: 0.61, 1.1), respectively.

## Discussion

Overall, this population-based case-control study of more than 100,000 women provided no evidence of an association between glucocorticoid use and breast cancer risk. The null association was consistent when dividing the use of glucocorticoids into recent and former use, intensity and duration of use, and also consistent with our previous study [10]. Our results answer the need for research on the role of glucocorticoids in breast cancer risk, which has not been well studied [11].

Our study has several strengths. The uniformly organized Danish healthcare system with complete hospital history and access to appropriate population controls allows a population-based case-control design. The use of population-based prescription registries, with a completeness approaching 100% [12,14] ensured unbiased assessment of exposure data preceding breast cancer diagnosis and the registry source eliminated recall bias. In all our analyses, we included only persons with at least 5 years of prescription history, thus potential bias due to left censoring of exposure information was reduced compared with the earlier study [10].

Our study also had limitations. We had no data regarding adherence with prescriptions, potentially leading to non-differential misclassification of some non-users as users. However, our drug exposure assessment was based on redeemed prescriptions, and because patients had to pay a proportion of the drug cost, our estimates are likely to reflect actual use, especially in women with more than two prescriptions. In support of this expectation, a validation study on postmenopausal HRT by Danish women showed good agreement between self-reported use and prescription data in the registry [24]. We also had no information on body mass index, alcohol consumption or family history of breast cancer, and other factors that may impact the risk of breast cancer [25]. To confound our results substantially they would also have to be related to glucocorticoid use conditional on adjustment for the measured covariates, which we have no reason to expect.

A more important limitation might be our inability to examine the impact of glucocorticoid use on breast cancer risk by specific breast cancer characteristics such as hormone receptor status. Studies have addressed the possibility of cross-talk between estrogen receptors and glucocorticoid receptors in mammary epithelial cells [26] and recently *in vivo *studies have suggested that glucocorticoids may stimulate the expression of the sulfotransferase SULT1E1, which plays a role in deactivating estrogen [27]. Thus, estrogen levels and estrogen receptor expression might well impact the action of glucocorticoids. Glucocorticoid response may also vary due to glucocorticoid receptor gene polymorphisms. The increased risk of squamous cell carcinoma in glucocorticoid users is more pronounced in the presence of the allele with the common genetic variant in the glucocorticoid receptor gene, compared with the homozygote wild types [28] but we were unable to evaluate this gene-drug interaction. Finally, glucocorticoids increase the expression of insulin-like growth factor-1 (IGF-1) receptors [29] and the levels of circulating IGF-1 [30]. IGF-1 receptor expression patterns in epithelial cells of normal breast tissue biopsies were associated with an increased risk of subsequent breast cancer [31] and a meta-analysis from 2010 concluded that circulating IGF-1 levels are positively related to estrogen-receptor-positive breast tumors, regardless of menopausal status [32]. Our study did not, however, have measurements of IGF-1 receptor or circulating IGF-1 available for analyses.

## Conclusions

There are several mechanisms by which glucocorticoid prescriptions might affect breast cancer risk in subpopulations defined by molecular subcharacteristics. Evaluations of these associations would require a study with detailed biological data. Overall, however, our results provide no evidence of an increased risk of breast cancer in glucocorticoid users compared with never users.

## Abbreviations

aOR: adjusted odds ratio; ATC: Anatomical Therapeutic Chemical; CI: confidence interval; COPD: chronic obstructive pulmonary disease; CPR number: civil personal registration number; CRS: Civil Registration System; DCR: Danish Cancer Registry; DNPR: Danish National Patient Registry; GR: glucocorticoid receptor; IBD: inflammatory bowel disease; ICD: International Classification of Diseases; IGF-1: insulin-like growth factor-1; HRT: hormone replacement therapy; OR: odds ratio; RA: rheumatoid arthritis.

## Competing interests

None of the authors received any fees, honoraria, grants or consultancies that would constitute a conflict of interest with the current study. The Department of Clinical Epidemiology, Aarhus University Hospital, receives funding for other studies from pharmaceutical companies in the form of research grants to (and administered by) Aarhus University. None of these studies have any relation to the present study.

## Authors' contributions

GVS designed the study, performed statistical analysis, interpreted the data, and drafted the manuscript. TLL conceived of the study and its design, provided epidemiological expertise, contributed to the interpretation of the data and critically revised the manuscript and contributed to major revisions. HTS and DCF conceived the study, and participated in its design, interpretation of data, and critically revised the manuscript. LP and SPU provided statistical expertise, analyzed the data, contributed to the interpretation of the data and critically reviewed the manuscript. All authors have read and approved the manuscript for publication.

## Supplementary Material

Additional file 1 **List of primary exposure drugs, cancer diagnosis, potential confounder drugs and diseases, and associated ICD and ATC codes**. This file contains ATC codes for the primary exposure drugs and potential confounder drugs, ICD codes for the cancer diagnosis used, and ICD hospital diagnosis codes for the potentially confounder diseases. Click here for file

Additional file 2 **Cumulative prednisolone equivalent dose calculation and list of systemic glucocorticoids with associated prednisolone conversion factors**. This file contains a list of the prescribed systemic glucocorticoids during the study period, their associated prednisolone conversion factor used for the calculation, and presents how we calculated cumulative prednisolone equivalent doses. Click here for file

## References

[B1] Kotsopoulos J Chen WY Gates MA Tworoger SS Hankinson SE Rosner BA Risk factors for ductal and lobular breast cancer: results from the nurses' health study Breast Cancer Res 2010 12 R106 10.1186/bcr2790 21143857 PMC3046451

[B2] Schimmer BP Parker KL Brunton LL, Lazo JS, Parker KL Adrenocorticotropic hormone; adrenocortical steroids and their synthetic analogs; inhibitors of the synthesis and actions of adrenocortical hormones Goodman and Gilman's the Pharmacological Basis of Therapeutics 2006 New York: McGraw-Hill Medical Publishing Division 1587 1612

[B3] Wintermantel TM Bock D Fleig V Greiner EF Schütz G The epithelial glucocorticoid receptor is required for the normal timing of cell proliferation during mammary lobuloalveolar development but is dispensable for milk production Mol Endocrinol 2005 19 340 349 15471946 10.1210/me.2004-0068

[B4] Schacke H Docke WD Asadullah K Mechanisms involved in the side effects of glucocorticoids Pharmacol Ther 2002 96 23 43 10.1016/S0163-7258(02)00297-8 12441176

[B5] Schreiber RD Old LJ Smyth MJ Cancer immunoediting: integrating immunity's roles in cancer suppression and promotion Science 2011 331 1565 1570 10.1126/science.1203486 21436444

[B6] Mahmoud SM Paish EC Powe DG Macmillan RD Grainge MJ Lee AH Ellis IO Green AR Tumor-infiltrating CD8+ lymphocytes predict clinical outcome in breast cancer J Clin Oncol 2011 29 1949 1955 10.1200/JCO.2010.30.5037 21483002

[B7] Novosyadlyy R Lann DE Vijayakumar A Rowzee A Lazzarino DA Fierz Y Carboni JM Gottardis MM Pennisi PA Molinolo AA Kurshan N Mejia W Santopietro S Yakar S Wood TL LeRoith D Insulin-mediated acceleration of breast cancer development and progression in a non-obese model of type 2 diabetes Cancer Res 2010 70 741 751 10.1158/0008-5472.CAN-09-2141 20068149 PMC2946167

[B8] Michels KB Solomon CG Hu FB Rosner BA Hankinson SE Colditz GA Manson JE Type 2 diabetes and subsequent incidence of breast cancer in the Nurses' Health Study Diabetes Care 2003 26 1752 1758 10.2337/diacare.26.6.1752 12766105

[B9] Gunter MJ Hoover DR Yu H Wassertheil-Smoller S Rohan TE Manson JE Li J Ho GY Xue X Anderson GL Kaplan RC Harris TG Howard BV Wylie-Rosett J Burk RD Strickler HD Insulin, insulin-like growth factor-I, and risk of breast cancer in postmenopausal women J Natl Cancer Inst 2009 101 48 60 19116382 10.1093/jnci/djn415 PMC2639294

[B10] Sørensen HT Mellemkjær L Skriver MV Lash TL Olsen JH Baron JA No excess risk of breast cancer among female users of systemic glucocorticoids Cancer Epidemiol Biomarkers Prev 2005 14 1022 1023 10.1158/1055-9965.EPI-04-0488 15824184

[B11] Vaidya JS Baldassarre G Thorat MA Massarut S Role of glucocorticoids in breast cancer Curr Pharm Des 2010 16 3593 3600 10.2174/138161210793797906 20977423

[B12] Ehrenstein V Antonsen S Pedersen L Existing data sources for clinical epidemiology: Aarhus University Prescription Database Clinical Epidemiol 2010 2 273 279 10.2147/CLEP.S13458 PMC2998817 21152254

[B13] Pedersen CB Gøtzsche H Møller JO Mortensen PB The Danish Civil Registration System. A cohort of eight million persons Dan Med Bull 2006 53 441 449 17150149

[B14] Gaist D Sørensen HT Hallas J The Danish prescription registries Dan Med Bull 1997 44 445 448 9377907

[B15] Storm HH Michelsen EV Clemmensen IH Pihl J The Danish Cancer Registry-history, content, quality and use Dan Med Bull 1997 44 535 539 9408738

[B16] Jensen AR Overgaard J Storm HH Validity of breast cancer in the Danish Cancer Registry. A study based on clinical records from one county in Denmark Eur J Cancer Prev 2002 11 359 364 10.1097/00008469-200208000-00007 12195162

[B17] Frank L Epidemiology. When an entire country is a cohort Science 2000 287 2398 2399 10.1126/science.287.5462.2398 10766613

[B18] Barel V Breast cancer and hormone-replacement therapy in the Million Women Study Lancet 2003 362 419 427 12927427 10.1016/s0140-6736(03)14065-2

[B19] Bosco JLF Antonsen S Sørensen HT Pedersen L Lash TL Metformin and incident breast cancer among diabetic women: a population-based case-control study in Denmark Cancer Epidemiol Biomarkers Prev 2011 20 101 111 10.1158/1055-9965.EPI-10-0817 21119073

[B20] Andersen TF Madsen M Jorgensen J Mellemkjoer L Olsen JH The Danish National Hospital Register. A valuable source of data for modern health sciences Dan Med Bull 1999 46 263 268 10421985

[B21] Christensen K Schmidt MM Vaeth M Olsen J Absence of an environmental effect on the recurrence of facial-cleft defects N Engl J Med 1995 333 161 164 10.1056/NEJM199507203330305 7791818

[B22] Jacobs JWG Bjjlsma JWJ Firestein GS, Budd RC, Harris ED, McInnes IB, Ruddy S, Sergent JS Glucocorticoid therapy Kelly's Textbook of Rheumatology, Sauders 2008 8 865

[B23] Rothman KJ Greenland S Lash TL Modern Epidemiology 2008 3 Philadelphia, PA: Lippincott Williams & Wilkins

[B24] Løkkegaard EL Johnsen SP Heitmann BL Stahlberg C Pedersen AT Obel EB Hundrup YA Hallas J Sørensen HT The validity of self-reported use of hormone replacement therapy among Danish nurses Acta Obstet Gynecol Scand 2004 83 476 481 15059162 10.1111/j.0001-6349.2004.00376.x

[B25] Colditz GA Baer HJ Tamimi RM Schottenfeld D, Fraumeni JF Breast Cancer Cancer Epidemiology and Prevention 2009 3 New York: Oxford University Press, Inc.; Print publication date: 2006, Published to Oxford Scholarship Online: September

[B26] Kinyamu HK Archer TK Estrogen receptor-dependent proteasomal degradation of the glucocorticoid receptor is coupled to an increase in mdm2 protein expression Mol Cell Biol 2003 23 5867 5881 10.1128/MCB.23.16.5867-5881.2003 12897156 PMC166332

[B27] Gong H Jarzynka MJ Cole TJ Lee JH Wada T Zhang B Gao J Song WC DeFranco DB Cheng SY Xie W Glucocorticoids antagonize estrogens by glucocorticoid-receptor mediated activation of estrogen sulfotransferase Cancer Res 2008 68 7386 7393 10.1158/0008-5472.CAN-08-1545 18794126 PMC6551207

[B28] Patel AS Karagas MR Perry AE Spencer SK Nelson HH Gene-drug interaction at the glucocorticoid receptor increases risk of squamous cell skin cancer J Invest Dermatol 2007 127 1868 1870 10.1038/sj.jid.5700798 17392827

[B29] Van den Berg HW Claffie D Boylan M McKillen J Lynch M McKibben B Expression of receptors for epidermal growth factor and insulin-like growth factor I by ZR-75-1 human breast cancer cell variants is inversely related: the effect of steroid hormones on insulin-like growth factor I receptor expression Br J Cancer 1996 73 477 481 10.1038/bjc.1996.84 8595162 PMC2074471

[B30] Prummel MF Wiersinga WM Oosting H Endert E The effect of long-term prednisone treatment on growth hormone and insulin-like growth factor-1 J Endocrinol Invest 1996 19 620 623 8957747 10.1007/BF03349028

[B31] Tamimi RM Colditz GA Wang Y Collins LC Hu R Rosner B Irie HY Connolly JL Schnitt SJ Expression of IGF1R in normal breast tissue and subsequent risk of breast cancer Breast Cancer Res Treat 2011 128 243 250 10.1007/s10549-010-1313-1 21197570 PMC3116083

[B32] Key TJ Appleby PN Reeves GK Roddam AW Insulin-like growth factor 1 (IGF1), IGF binding protein 3 (IGFBP3), and breast cancer risk: pooled individual data analysis of 17 prospective studies Lancet Oncol 2010 11 530 542 20472501 10.1016/S1470-2045(10)70095-4 PMC3113287

