# Supplementary material for: Use of glucocorticoids and risk of breast cancer: a Danish population-based case-control study
Source: Breast Cancer Res. 2012 Feb 3;14(1):R21. doi: 10.1186/bcr3106 (PMC3496139; doi:10.1186/bcr3106)
Supplement: Additional file 1 — List of primary exposure drugs, cancer diagnosis, potential confounder drugs and diseases, and associated ICD and ATC codes. This file contains ATC codes for the primary exposure drugs and potential confounder drugs, ICD codes for the cancer diagnosis used, and ICD hospital diagnosis codes for the potentially confounder diseases. [file bcr3106-S1.PDF]

Additional file 1. List of primary exposure drugs, potentially confounder drugs, cancer diagnosis, potentially confounder diseases and associated ICD and ATC codes

Primary exposure drugs available and prescribed during study period (ATC codes)

Systemic glucocorticoids

- Betamethasone (H02AB01)
- Dexamethasone (H02AB02)
- Methylprednisolone (H02AB04)
- Prednisolone (H02AB06)
- Prednisone (H02AB07)
- Triamcinolone (H02AB08)
- Hydrocortisone (H02AB09)
- Cortisone (H02AB10)

Inhaled glucocorticoids

- Beclomethason (R03BA01)
- Budesonide (R03BA02)
- Flunisolid (R03BA03)
- Fluticasone (R03BA05)
- Mometason (R03BA07)

Local-acting glucocorticoids with intestinal anti-inflammatory effect

- Prednisolone (A07EA01)
- Hydrocortisone (A07EA02)
- Budesonide (A07EA06)

Potentially confounder drugs (ATC codes)

Postmenopausal hormone replacement therapy:

- Estrogens (G03C)
- Estrogens and progesterone combined (G03F)

Anti-diabetic medicine:

- Insulin and insulin analogs (A10A)
- Oral anti-diabetic medications (A10B–A10X) (minus metformin)
- Metformin (A10BA02)

Immunosuppressive medications (L04)

Cancer codes obtained from the Danish Cancer Registry

Breast cancer:
ICD-10: C50.0-50.6, C50.8 & C50.9

Other malignant cancers:
ICD-10: C00–C97 (minus non-melanoma skin cancer: ICD-10: C440–C449)

Hospital diagnose codes on potentially confounder diseases obtained from the Danish National Patient Registry

Rheumatoid arthritis
ICD-8: 712.19, 712.29, 712.39, 712.59
ICD-10: M05−M06, G73.7D, I32.8A, I39.8E, I41.8A, I52.8A

Chronic obstructive pulmonary disease
ICD-8: 490−492
ICD-10: J40−J44, J47

Asthma
ICD-8: 493
ICD-10: J45−J46

Obesity
ICD-8: 277.99
ICD-10: E66

Inflammatory bowel disease (ulcerative colitis or Crohn's disease)
ICD-8: 563.01, 563.19, 569.04
ICD-10: K50.0, K50.1, K50.8, K50.9, K51.0−K51.9

Diabetes
ICD-8: 249−250
ICD-10 codes E10−E11

Other autoimmune diseases:

Hematological system
Autoimmune hemolytic anemia
ICD-8: 283.90
ICD-10: D59.0, D59.1
Idiopathic thrombocytopenic purpura
ICD-8: 287.10

ICD-10: D69.3

Endocrine system

Autoimmune thyroiditis

ICD-8: 244.01, 245.03

ICD-10: E06.3

Addison´s disease

ICD-8: 255.10

ICD-10: E27.1

Grave's disease

ICD-8: 242.00, 242.01, 242.08, 242.09

ICD-10: E05.0

Central nervous / neuromuscular system

Multiple sclerosis

ICD-8: 340

ICD-10: G35

Myasthenia gravis

ICD-8: 733.09

ICD-10: G70.0

Gastrointestinal/hepatobilliary system

Pernicious anemia

ICD-8: 281.00, 281.01, 281.08, 281.09

ICD-10: D51.0

Coelic disease

ICD-8: 269.00

ICD-10: K90.0

Primary biliary cirrhosis

ICD-8: 571.90

ICD-10: K74.3

Skin

Atopic dermatitis

ICD-8: 691.00

ICD-10: L20

Pemphigus/pemphigoid

ICD-8: 694.00-694.03, 694.05

ICD-10: L10.0, L10.1, L10.2, L10.4, L12.0

Dermatitis herpetiformis

ICD-8: 693.08, 693.09

ICD-10: L13.0

Psoriasis

ICD-8: 696.09, 696.10, 696.19

ICD-10: L40, M07.0–M07.3

Connective tissue diseases

Ankylosing spondylitis

ICD-8: 712.49

ICD-10: M45, H221B

Polymyositis/dermatomyositis
ICD-8: 716.09, 716.19
ICD-10: M33
Systemic- and subacute cutaneous lupus erythematosus
ICD-8: 734.19
ICD-10: M32, G05.8A, G73.7C, I32.8B, I39.8C, L93.1, L93.2, N08.5A, N16.4B
Systemic scleroderma
ICD-8: 734.00–734.09
ICD-10: M34.0–34.9
Mixed connective tissue disease
ICD-10: M35.1
Sjögren´s syndrome
ICD-8: 734.90
ICD-10: M35.0, G73.7A, N16.4A
Sarcoidosis
ICD-8: 135.99
ICD-10: D86, G53.2, H22.1A, I41.8B, K77.8B, M63.3
Vasculitis syndromes including polymyalgia rheumatic
ICD-8: 287.09, 446.09–446.99
ICD:10: D69.0B, I77.6, L95, M30–M31, M35.3, M35.6, M79.3, N08.5B–N08.5E

Abbreviations

ATC: Anatomical Therapeutic Chemical; ICD-8: International Classification of Diseases, Eighth Revision; ICD-10: International Classification of Diseases, Tenth Revision.
