# Supplementary material for: Use of glucocorticoids and risk of breast cancer: a Danish population-based case-control study
Source: Breast Cancer Res. 2012 Feb 3;14(1):R21. doi: 10.1186/bcr3106 (PMC3496139; doi:10.1186/bcr3106)
Supplement: Additional file 2 — Cumulative prednisolone equivalent dose calculation and list of systemic glucocorticoids with associated prednisolone conversion factors. This file contains a list of the prescribed systemic glucocorticoids during the study period, their associated prednisolone conversion factor used for the calculation, and presents how we calculated cumulative prednisolone equivalent doses. [file bcr3106-S2.PDF]

Additional file 2. Cumulative prednisolone equivalent dose calculation and list of systemic glucocorticoids with associated prednisolone conversion factors

Systemic glucocorticoid	Equivalent glucocorticoid dose (mg)[a]	Prednisolone conversion factor (PCF)
Cortisone	25	0.20
Hydrocortisone	20	0.25
Methylprednisolone	4	1.25
Prednisolone	5	1
Prednisone	5	1
Triamcinolone	4	1.25
Dexamethasone	0.75	6.67
Betamethasone	0.60	8.33

[a] Based on the equivalency table in *Kelly's Textbook of Rheumatology* [22].

Cumulative dose calculation:

The cumulative dose was obtained by multiplying number of pills/injections x dose per pill/injection x PCF for each prescription and then summing across all prescriptions.
